# Impact of Ultrasound-Guided Peripheral Intravenous Training on Central Line Placement in the Emergency Department

**DOI:** 10.5811/westjem.58319

**Published:** 2026-09-09

**Authors:** Matthew Berniard, Richard Slama, Gavin Rogers, Elena Garrett, Chandler Davis, John Alex

**Affiliations:** Riverside Regional Medical Center, Department of Emergency Medicine, Newport News, Virginia

## Abstract

**Introduction:**

Central venous catheters are placed in the emergency department (ED) when difficult peripheral access limits care or when critical medications require central access because of their sclerotic nature or incompatibility with other drugs. Ultrasound-guided peripheral intravenous (IV) access offers a less invasive alternative. We evaluated whether implementation of a structured ultrasound-guided peripheral intravenous training program for ED nurses and technicians was associated with changes in ED central venous catheter use.

**Methods:**

We performed a retrospective chart review at our 50-bed academic community ED, which holds a Level II trauma designation. This center has an ED census of 80,000 patients per year, a 4:1 nursing to patient ratio with an admission rate of 20–25% with expected monthly variation. A structured ultrasound-guided peripheral IV curriculum for nurses and technicians launched in November 2023. The primary endpoint was annual ED central venous catheter placement count, identified through procedure documentation in the electronic health record. Training that was conducted for nurses and clinical technicians consisted of three hours of didactic and hands-on training, followed by a period of procedural supervision. We compared the baseline year 2022 with the first two full post-implementation years, 2024 and 2025. The analysis was descriptive.

**Results:**

Central venous catheter placements in the ED decreased from 387 in 2022 to 275 in 2024 and 218 in 2025, corresponding to absolute reductions of 112 placements (28.9%) and 169 placements (43.7%), respectively. By the end of 2025, 33 nurses and ED technicians had completed training and competency sign-off.

**Conclusion:**

Implementation of a structured ultrasound-guided peripheral IV training program for nurses and technicians was associated with fewer ED central venous catheter placements at our institution. These findings support further multicenter evaluation using patient-level outcomes and visit-adjusted rates.

## INTRODUCTION

Establishing vascular access is fundamental to emergency care. In patients with difficult intravenous (IV) access, repeated failed peripheral attempts can lead to escalation to physician-performed ultrasound-guided peripheral IV (USPIV) placement or, in selected cases, central venous catheter (CVC) placement. Although CVCs can be critical to patient care, it is invasive and carries meaningful mechanical, infectious, and thrombotic risk.^1^,^2^

Ultrasound-guided peripheral IV placement improves vascular access success in difficult-access patients and can provide an alternative to more invasive access strategies.^4^ Prior studies have also shown that nurse- and technician-performed USPIV can be taught successfully in emergency and hospital settings.^5^–^8^ At our institution, difficult-access escalation historically depended on clinician availability and operator comfort with bedside ultrasound. Expanding USPIV capability to nurses and technicians offered a plausible systems-level mechanism to reduce avoidable CVC placement for access alone. The objective of this study was to investigate whether an association between implementation of a structured USPIV training and competency program for emergency department (ED) nurses and technicians and subsequent ED CVC placement counts existed.

## METHODS

We performed a retrospective chart review at our 50-bed academic community hospital with Level II trauma designation and an annual ED census of approximately 80,000 visits. The investigation was designed as an operational quality-improvement evaluation of a vascular access training initiative rather than a hypothesis-testing study.

The USPIV training program launched in November 2023. The curriculum included didactic instruction, simulation, supervised bedside scanning and cannulation, and competency sign-off by ultrasound-trained emergency medicine faculty. Monthly classes were offered during the implementation period. A total of 33 nurses and ED technicians completed training and competency by the end of 2025. Because exact monthly attendance counts were not retained, training volume is reported cumulatively rather than month by month. In the ED at our hospital, a 4:1 nursing ratio is maintained; and by the end of 2025, 20.6% of the department’s nursing and technical workforce were competent in the USPIV procedure.[Fig f1-wjem-27-1447]

**Population Health Research Capsule**

What do we already know about this issue?

*Ultrasound-guided peripheral intravenous (USPIV) placement can improve access in difficult-IV patients and may reduce use of more invasive vascular access*.

What was the research question?

*Was nurse/technician USPIV training associated with fewer emergency department (ED) central lines?*

What was the major finding of the study?

*Placememt of central lines in the ED fell 43.7% after training: 387 in 2022 vs 218 in 2025*.

How does this improve population health?

*Expanding frontline USPIV may reduce invasive central line use, thereby lowering complication risks and potentially saving about $136,000 annually*.

The program was available within a department that also supports physician and advanced practice practitioner ultrasound education; however, this initiative primarily emphasized bedside nurses and ED technicians because they perform the earliest vascular access attempts in local workflow. Independent competency required successful completion of the curriculum and supervised clinical sign-off. The threshold for independent procedural competency was 10 supervised USPIVs; this threshold was retained as the operational standard during rollout.

The primary endpoint was the annual number of ED CVC placements. Data were extracted from the Epic electronic health record system (Epic Systems Corp). using the SlicerDicer function. Queries were limited to procedure documentation attributable to ED attending and resident physicians to avoid misclassification from lines placed by non-ED services in boarded patients. The 2022 calendar year served as the baseline preintervention period. The 2023 implementation year was excluded from the primary pre/post comparison because only November and December were exposed to the intervention. The first and second full postimplementation years were 2024 and 2025.^3^

This retrospective analysis was structured according to the guidelines for retrospective chart review outlined by Worster et al.^3^ Analyses were descriptive only. Table 1 summarizes the methodological criteria and their corresponding descriptions relevant to this analysis, as adapted from Worster et al.^3^ Because available data consisted of aggregated administrative procedure counts without patient-level covariates, indication-level adjudication, or consistently available ED-visit denominators for rate calculation, we did not perform formal inferential testing or interrupted time-series modeling. Absolute and relative differences from the baseline are reported.

## RESULTS

In the baseline year 2022, we identified 387 CVC placements in the ED, After the November 2023 program launch, the first full post-implementation year (2024) included 275 placements, an absolute reduction of 112 procedures and a relative reduction of 28.9% from baseline. In 2025, 218 CVC placements in the ED were identified, an absolute reduction of 169 procedures and a relative reduction of 43.7% from baseline. Month-by-month counts for 2022, 2024, and 2025 are shown in Figure 2 to illustrate the temporal distribution across study years.

By the end of 2025, 33 nurses and ED technicians had completed USPIV training and competency. There appeared to be an increase in USPIV documentation after implementation in ancillary reports reviewed during manuscript development; however, because USPIV procedure capture was incomplete and inconsistent across clinician groups, USPIV counts were not analyzed as a formal secondary endpoint.

## DISCUSSION

In this single-center retrospective analysis, implementation of a structured USPIV training and competency program for ED nurses and technicians was associated with a sustained decrease in annual ED CVC placement counts over the subsequent two full calendar years. Because the study endpoint was aggregate CVC use rather than patient-level access indication, our findings are best interpreted as a systems-level association rather than proof of causation.

These findings are directionally consistent with prior studies showing that nonphysician USPIV training can reduce reliance on more invasive vascular access devices. Galen et al reported decreased use of peripherally inserted central catheters and midline catheters after nurse USPIV training on an inpatient unit; and Amick et al found reduced midline use after a hospital-wide simulation-based mastery learning curriculum for inpatient nurses.^9^,^10^

The emergency medicine literature also supports the feasibility of nurse- and technician-performed USPIV. Earlier studies demonstrated successful emergency nurse USPIV adoption and technician training with physician-level success rates, supporting the plausibility that broader frontline USPIV capability can change escalation patterns for difficult vascular access.^5^–^7^ The present study extends that literature by focusing specifically on CVC use in the ED after a nurse- and technician-focused training initiative. In local workflow, difficult access previously escalated to physician involvement when bedside attempts failed. Increasing frontline USPIV capability plausibly reduced the need for escalation to physician-performed invasive access in at least some patients whose primary barrier was vascular access rather than a true central-access indication.[Table t2-wjem-27-1447]

As with the implementation of any clinical QI initiative, this program was associated with modest upfront costs. Importantly, these costs were minimized by leveraging the existing infrastructure of our emergency ultrasound program, which includes dedicated emergency ultrasound faculty, an established ultrasound service line, fellowship leadership, a residency director, and two active fellows. Development of asynchronous educational content was completed as part of routine emergency ultrasound faculty educational responsibilities and supported through existing stipends. Live didactic instruction, simulation-based training, and initial physician-supervised clinical practice were similarly incorporated into established physician educational and clinical compensation models and did not generate additional direct institutional costs.

Nursing staff participating in in-person training were compensated at their standard hourly rate for three hours of training, inclusive of initial supervised clinical practice, at an average rate of $49 per hour (range, $38-$61). Additional supervised clinical practice required to achieve competency occurred during regularly scheduled nursing shifts and did not result in incremental compensation beyond existing staffing costs. Technicians were compensated under the same training time framework at an average hourly rate of $22 (range, $19-$24). Accordingly, the mean direct training cost was $148 per nurse and $65 per ED technician. Based on 33 trained staff by the end of 2025, the total direct training cost was $3,974, which corresponds to approximately $1,325 per year when annualized across the initial three-year implementation period. Using our hospital’s estimated direct cost of $811 per ED CVC placement and the observed reduction of 169 CVCs in 2025 compared with the 2022 baseline, a simple program-level estimate suggests $137,132 in gross annual avoided CVC cost. After subtracting the annualized training expense, this corresponds to an approximate net annual savings of $135,807.[Table t3-wjem-27-1447]

This estimate assumes that the observed reduction in CVC use was attributable to the training program and that many avoided CVCs were replaced by lower cost peripheral access pathways, most commonly USPIV, rather than reflecting case-mix changes alone. It also does not include potential downstream savings from avoidance of CVC-related complications, which may be substantial and further improve the economic value of the program. Taken together, these findings suggest that this program can be implemented in a cost-conscious, scalable, and financially favorable manner within institutions possessing established ultrasound infrastructure.

Importantly, we did not measure access-related complications, time to treatment, length of stay, patient satisfaction, or direct costs; those outcomes should not be inferred from the present data. Future work should prioritize rate-based analyses using ED visit denominators, patient-level review of CVC indications, and downstream outcome measures such as complications, throughput, and patient experience.

## LIMITATIONS

This study has several limitations. First, it was conducted at a single institution with an established emergency ultrasound infrastructure, which may limit generalizability. Second, the retrospective design and reliance on administrative procedure documentation create risk of missing or misclassified data. Third, the primary analysis used aggregated annual counts without patient-level covariates, limiting adjustment for secular trends or other concurrent practice changes. Fourth, we did not have consistently available ED-visit denominators for rate calculation; therefore, the results are reported as counts rather than rates per 1,000 visits. Fifth, we could not reliably adjudicate which CVCs were placed exclusively for difficult peripheral access versus other clinical indications. Finally, USPIV procedure documentation was not sufficiently complete across clinician groups to support a robust secondary endpoint analysis.

## CONCLUSION

Implementation of a structured USPIV training and competency program for ED nurses and technicians was associated with a sustained reduction in ED CVC placement counts at our institution. These findings support further study of non-physician USPIV capability as a strategy to reduce potentially avoidable invasive vascular access while preserving appropriate central access for patients who truly need it.

## Figures and Tables

**Figure 1 f1-wjem-27-1447:**
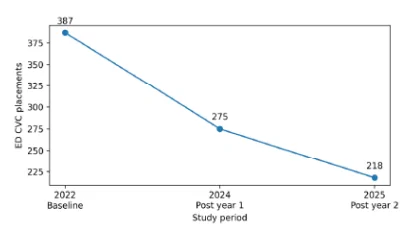
Number of central venous catheter placements in the emergency department annually across study periods before and after the rollout of an ultrasound-guided peripheral intravenous placement program. The 2023 rollout year was excluded from the primary comparison due to November 2023 program launch date. *CVC*, central venous catheter*; ED*, emergency department.

**Figure 2:**
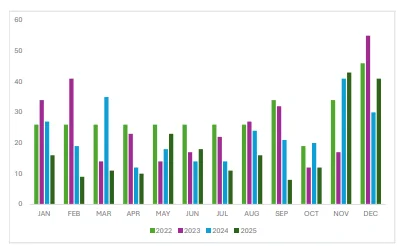
Month-by-month emergency department central venous catheter placements in 2022, 2024, and 2025. *CVC*, central venous catheter.

**Table 1:** Methodological features of a retrospective chart review that evaluated whether a structured ultrasound-guided peripheral intravenous training program for nurses was associated with changes in use of central venous catheter use in the emergency department.

Methodologic Element	Description
Study design	Retrospective descriptive chart review / quality-improvement evaluation
Setting	Single academic community ED, Level II trauma center, ~80,000 annual visits
Intervention	Structured USPIV training and competency program for ED nurses and technicians launched November 2023
Primary endpoint	Annual number of ED CVC placements
Data source	Epic EHR queried with SlicerDicer
Inclusion	ED CVC procedure documentation attributed to ED attending and resident physicians
Exclusion	Central lines placed by non-ED services in boarded/admitted patients
Analytic periods	2022 baseline; 2024 and 2025 full post-implementation years; 2023 rollout year excluded from primary comparison

*CVC*, central venous catheter; *ED*, emergency department; *EHR*, electronic health record; *USPIV*, ultrasound-guided peripheral intravenous.

**Table 2 t2-wjem-27-1447:** Annual central venous catheter placements in the emergency department before and after implementation of an ultrasound-guided peripheral intravenous placement program.

Year	Study Phase	ED CVC Placements	Change vs. 2022 Baseline
2022	Preintervention baseline	387	Reference
2023	Rollout year (November-December exposed)	Excluded from primary comparison	Partial implementation year
2024	First full postimplementation year	275	−112 (−28.9%)
2025	Second full postimplementation year	218	−169 (−43.7%)

*CVC*, central venous catheter; *ED*, emergency department.

**Table 3 t3-wjem-27-1447:** Central venous line complication rates and associated cost.^11^–^14^

Complication	Rate per 100 Lines Placed	Cost Estimate
Infection (CLABSI)	3	$16,000–$29,000
Thrombosis	5	$10,000 – $25,000
Pneumothorax	1	$5,000 – $20,000
Death	0.5	$50,000 – $200,000

*CLABSI*, central line-associated bloodstream infection.
